# Benchmarking the impact of information processing in the insect olfactory system with a spiking neuromorphic classifier

**DOI:** 10.1186/1471-2202-12-S1-P233

**Published:** 2011-07-18

**Authors:** Michael Schmuker, Chris Häusler, Daniel Brüderle, Martin P Nawrot

**Affiliations:** 1 Neuroinformatics & Theoretical Neuroscience, Institute of Biology, Freie Universität Berlin, Berlin, Germany; 2 Kirchhoff Institute for Physics, Heidelberg University, 69120 Heidelberg, Germany; 3 Bernstein Center for Computational Neuroscience Berlin, 10119 Berlin, Germany

## 

A large body of behavioral discrimination experiments demonstrates that honeybees can identify odorant stimuli in a quick and reliable fashion [1]. The neuronal circuits involved in odor discrimination are well described at a structural level. In this contribution, we decomposed the honeybee’s olfactory pathway into successive processing stages, implemented them in a spiking neuronal network model and benchmarked the contribution of each stage to the performance of a neuronal implementation of a probabilistic classifier.

In the insect olfactory system, primary receptor neurons project to the antennal lobe (AL). The AL is organized in compartments called glomeruli, with each glomerulus receiving input only from one type of receptor neurons. Each type of receptor neuron/glomerulus can be seen as gradually encoding the presence of a certain feature of the stimulus. Strong lateral inhibitory interactions between glomeruli make an impact on information processing and decrease correlation between feature channels [3,4]. Uniglomerular projection neurons (PNs) send their axons from glomeruli in the AL to Kenyon cells (KCs) in the mushroom body (MB), a central brain structure where different sensory modalities converge and stimulus associations are formed [5]. Connections between PNs and KCs are realized within small local microcircuits, where PNs and KCs interact with an inhibitory cell population originating in the protocerebral-calycal tract (PCT neurons) [6].

Based on this architecture, we implemented a network of integrate-and-fire neurons which can be trained in a supervised fashion to discriminate classes in multidimensional input data. Input data patterns were converted into spike trains and fed into the AL stage, where we implemented lateral inhibitory interactions between glomeruli. In the MB stage, we implemented microcircuits in which excitatory PNs interact with inhibitory PCT neurons before projecting onto KCs, which in turn projected on a decision circuit that supports supervised training according to a biologically plausible learning rule [2].

We found that the performance of the decision circuit critically depended on the structure of the training data. The decision circuit performed well on non-overlapping stimulus classes that could be separated along the identity diagonal in feature space. However, the network failed to learn data configurations where stimulus classes overlapped, or where separation along the identity diagonal was not possible. We show how this limitation can be compensated for by the PN-PCT-KC circuits in the MB stage, which create non-linear transformations of the input data that enable the decision circuit to learn also those configurations requiring separation off the identity diagonal.

Currently, we extend our network to cope with data of arbitrary dimensionality, in order to test classification performance on benchmark data sets and a real-world odorant data set [4]. We are also porting our model network to neuromorphic hardware [7]. This is a first step towards implementations of fast and powerful neuromorphic classification devices, applicable to a wide range of parallel sensor data.
